# The potential for climate-driven bathymetric range shifts: sustained temperature and pressure exposures on a marine ectotherm, *Palaemonetes varians*

**DOI:** 10.1098/rsos.150472

**Published:** 2015-11-25

**Authors:** J. P. Morris, S. Thatje, D. Cottin, A. Oliphant, A. Brown, B. Shillito, J. Ravaux, C. Hauton

**Affiliations:** 1 Ocean and Earth Science, University of Southampton, European Way, Southampton SO14 3ZH, UK; 2 UPMC Université Paris 06, UMR-CNRS 7208, 7 Quai St-Bernard, Paris 75005, France

**Keywords:** hydrostatic pressure, temperature, physiological scope

## Abstract

Range shifts are of great importance as a response for species facing climate change. In the light of current ocean-surface warming, many studies have focused on the capacity of marine ectotherms to shift their ranges latitudinally. Bathymetric range shifts offer an important alternative, and may be the sole option for species already at high latitudes or those within enclosed seas; yet relevant data are scant. Hydrostatic pressure (HP) and temperature have wide ranging effects on physiology, importantly acting in synergy thermodynamically, and therefore represent key environmental constraints to bathymetric migration. We present data on transcriptional regulation in a shallow-water marine crustacean (*Palaemonetes varians*) at atmospheric and high HP following 168-h exposures at three temperatures across the organisms’ thermal scope, to establish the potential physiological limit to bathymetric migration by neritic fauna. We observe changes in gene expression indicative of cellular macromolecular damage, disturbances in metabolic pathways and a lack of acclimation after prolonged exposure to high HP. Importantly, these effects are ameliorated (less deleterious) at higher temperatures, and exacerbated at lower temperatures. These data, alongside previously published behavioural and heat-shock analyses, have important implications for our understanding of the potential for climate-driven bathymetric range shifts

## 1. Background

Global change events are profoundly altering biology [1–4]. Organisms occupying an environment where directional change is occurring must move, acclimatize or adapt in order to prevent extinction (acclimatization describes adjustments of phenotype not related to genome changes which occur within a single generation, whereas adaptation describes a change in genotype which requires multiple generations) [2,5]. Significant progress has been made in documenting latitudinal range shifts in response to environmental warming both in terrestrial [4] and marine habitats [6,7]. Recently, studies have shown that some marine ectotherms are undergoing bathymetric range shifts in response to warming surface waters [8–10]. Bathymetric range shifts may be an important [11,12] but typically overlooked alternative to latitudinal shifts, and share similar biotic and abiotic factors as marine latitudinal constraints with the addition of hydrostatic pressure (HP).

HP is a thermodynamic variable, and as such affects all biological processes [13]. It is of particular significance in the marine realm as pressure gradients are much steeper in water than in air. Consequently, the upper and lower depth distributions of marine organisms are delineated in part by HP [11]. Despite its significance, HP is rarely considered as a stressor in the marine environment in comparison to other factors such as temperature. Undoubtedly, research involving HP is diminutive in part due to technological limitations. Yet, research concerning the effects of acute elevated HP (2 h exposures) has shown that temperature changes can mediate the effects of HP [14].

This study explores whether the apparent effects of HP and temperature, observed in acute experimental exposures [14], are an artefact of rapid changes in these variables, or represent a truly ecologically relevant response. Such findings have implications on our understanding of depth distributions and range shifts of marine ectotherms. We have studied the transcriptional regulation of genes linked with the onset of a variety of pressure intolerances in a shallow-water shrimp, *Palaemonetes varians*, following a sustained hyperbaric exposure at three temperatures across the species’ thermal tolerance breadth. Behavioural analysis and the observation of changes in heat-shock response have been previously published from the same exposures in *P. varians* [15]. This study expands on previous observations by quantifying the expression of a pressure-specific stress marker, and several metabolism-related genes. Six genes were selected for expression analysis that had been previously shown to provide insight into the sub-lethal effects of elevated HP and changing temperature [14] and are summarized in table 1. The *narg* gene codes for an *N*-methyl-d-aspartate receptor (NMDAR)-regulated protein. Upregulation has been shown under elevated HP scenarios in both the shallow-water shrimp *P. varians* [14,21], and also a continental slope-depth king crab, *Lithodes maja* [22]. Elevated HP, beyond natural distribution limits, has been shown to cause neurophysiological disturbances such as spasming and convulsions in a number of organisms, from rats [23] to shrimp [24]. These disturbances are thought to be associated with NMDAR hyperactivity [16], which is hypothesized to be related to differential *narg* gene expression [17]. Thus, *narg* gene regulation can be thought of as a marker of pressure intolerances associated with neurophysiological disturbances and behavioural pathologies [14]. The *hsp70 f1* and *f2* genes code for 70 kDa heat-shock protein (HSP70) isoforms. HSP70s are molecular chaperones that show increased transcriptional regulation and protein activity under stressful scenarios that lead to intracellular macromolecular damage. Consequently, genes coding for HSP70s have been widely used as general markers of stress as they are key members of the near-ubiquitous cellular stress response (CSR) [18]. The *ldh* gene,*cs* gene and *gapdh* gene code for proteins involved in key metabolic pathways [19,20]. The *ldh* gene codes for the lactate dehydrogenase enzyme that catalyses the interconversion of pyruvate and lactate [25]. Increased levels of lactate have been shown to correlate with lactate dehydrogenase isozyme expression [26]. Expression of the *ldh* gene may therefore be a proxy for lactate accumulation and consequently anaerobic metabolism. Both the *gapdh* gene and the *cs* gene code for important members of aerobic metabolic pathways. The *gapdh* gene codes for the enzyme glyceraldehyde-3-phosphate dehydrogenase which catalyses the sixth step of glycolysis. The *cs* gene codes for the enzyme citrate synthase which is a key and rate-limiting member of the tricarboxylic acid cycle [19]. The expression of the *cs* gene has been recently shown to be correlated with mitochondrial citrate synthase activity [27,28], and the *gapdh* gene has shown increased expression during periods of elevated oxygen consumption in *P. varians* [21]. Although not clearly resolved, changes in the transcriptional regulation may have consequences for the abundance of the downstream proteins they encode, thus affecting aerobic metabolism.

**Table 1.** Short summary of the genes used, and their relevance to the study.

gene	relevance to this study	reference
*narg* gene	an NMDA-receptor regulated marker of HP intolerance associated with neurophysiological disturbances	[14,16,17]
*hsp70 isoforms* (*f*1 and *f*2)	encode heat-shock proteins; markers of cellular macromolecular damage associated with the generalized effects of cellular stress	[18]
*cs* gene	encodes an enzyme (citrate synthase) involved in a rate-limiting step in aerobic metabolism	[19]
*ldh* gene	encodes an enzyme (lactate dehydrogenase) involved in anaerobic metabolism	[20]
*gapdh* gene	encodes an enzyme (glyceraldehyde-3-phosphate dehydrogenase) involved in aerobic metabolism	[14,18]

The adult life-stage was chosen for this study as it probably represents the most sensitive stage to stress beyond very early life-stages [29], where a lack of tissue presents methodological challenges. A recent study conducted on *L. maja*, a mid-depth king crab, suggested a reduction in HP tolerance through ontogeny [22]. As such, the adult life-stage may be a particularly sensitive stage to changes in HP. For successful climate-driven bathymetric range shifts to occur, all life-stages must be able to tolerate increases in HP. Thus, in attempting to determine physiological limits to temperature and HP changes the adult life-stage was chosen as a potentially sensitive stage in the life cycle of *P. varians*.

## 2. Material and methods

Adult *P. varians* shrimp (4–5 cm total length) were collected from Lymington salt marshes (Hampshire, UK). The water temperature during collection was approximately 15°C. The shrimp were acclimated to 5, 10 or 27°C±0.5°C at a rate of 2°C day^−1^. Shrimp were maintained for a further 3 days at acclimation temperature before HP experiments.

The IPOCAMP™ system [30] was used to conduct week-long 168 h HP and temperature exposures. The 168 h exposure time was chosen as a step away from previous acute 2–6 h HP exposures, and represents a trade-off between length of exposure, the effects of starvation (a current technological limitation of the IPOCAMP™ system) and specimen mortality. Mortality was observed in the shrimp from 192 h of exposure onwards at 15°C and 10 MPa (JP Morris, A Brown, A Oliphant, D Cottin 2015, unpublished data), and therefore 168 h exposures represent a significant but sub-lethal stress scenario. The system was filled with aerated filtered seawater (salinity: approx. 32) and acclimated to either 5, 10 or 27°C±0.1°C. Shrimp were transferred into the hyperbaric chamber, and the system was set running at atmospheric pressure for 1 h before the start of each exposure, allowing some time for recovery from any minor handling stress experienced. HP was then increased stepwise, at a rate of 1 MPa every 5 min, up to 10 MPa (10 MPa; ≈1000 m depth). Shrimp were held under these conditions for 168 h; 0.1 MPa control treatments were run over the same time period at each temperature. After exposure, the system was depressurized over 1 min, and shrimp were snap frozen with liquid nitrogen for RNA extraction.

RNA extraction, DNase-treatment and reverse-transcription were conducted and all necessary quality control measures were met, according to Bustin *et al.* [31]. qPCR primers were designed and optimized in accordance with the MIQE guidelines [31]. Primer sequences, concentrations, linear dynamic ranges, reaction efficiencies and reference gene normalization strategies are listed in the electronic supplementary material. Assay specificity was confirmed by melt curve analysis. Normalized relative quantities (NRQs) were calculated using qBase+ software. NRQs were then scaled giving a value of relative fold change (RFC). RFC is a measure of relative changes in gene expression. The RFC of each gene was determined relative to the atmospheric control exposures at each temperature. Statistical significance of mean RFC was identified at *p*<0.05, determined by GLM and *post hoc* Tukey-HSD test.

## 3. Results

**Figure 1.**
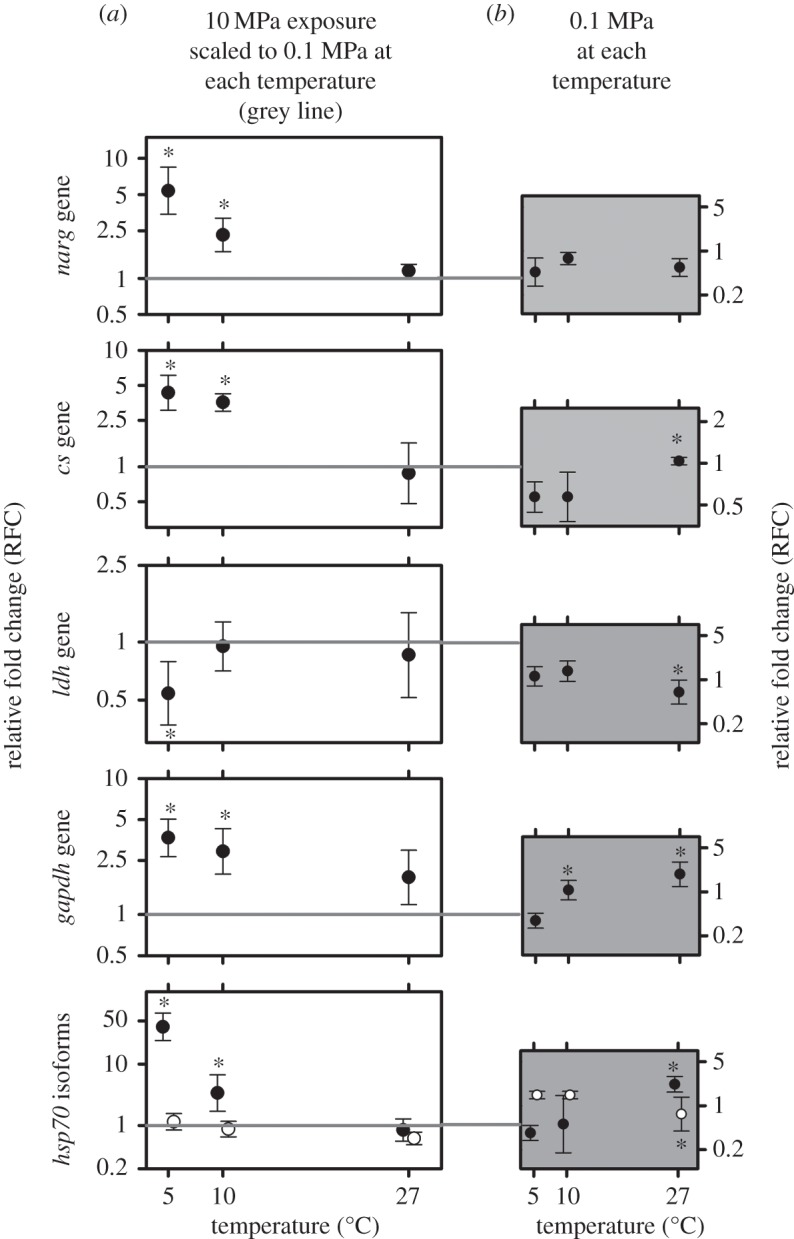
Relative fold change (RFC) of six genes after 168 h exposures at 0.1 MPa and 10 MPa at three temperatures: 5, 10 and 27°C. (*a*) RFCs of each named gene (*hsp70 f1* isoform—black dots, *hsp70 f2* isoform—white dots) at 10 MPa compared with 0.1 MPa at 5, 10 and 27°C. RFCs are scaled to gene expression at the corresponding temperature at 0.1 MPa, represented by the grey line in each graph. (*b*) Unscaled averaged RFCs at 0.1 MPa at 5, 10 and 27°C (represented by straight grey line in *a*). RFCs and 95% CIs calculated from five biological replicates. Significance displayed as *p<0.05 determined by a GLM and a *post hoc* Tukey-HSD test.

Relative fold change (RFC) of five genes showed significant differences under elevated HP across the three experimental temperatures (5, 10 and 27°C; figure 1*a*) when compared with atmospheric control treatments. To observe the effects of elevated HP at different temperatures, the RFC of each gene was determined relative to the atmospheric control exposures at each temperature. Further, the atmospheric control treatment was scaled to a RFC of 1 in each case (figure 1*a*). Consequently, the RFC values and figure 1*a* represent the effects of elevated HP on each gene, and how such an effect is influenced by different experimental temperatures. It is important to note that this study involved the quantification of relative expression, as explained in the methods section, not absolute expression. Thus, direct RFC comparisons between genes cannot be made. It is only correct to compare the RFCs and relative fold patterns within each gene across the different HPs and temperatures. Further, by scaling the atmospheric control treatments at each temperature to 1, temperature-only effects are removed from figure 1*a*. For transparency, these temperature-only effects have been shown in figure 1*b*.

The *narg* gene, the *cs* gene, the *gapdh* gene and the *hsp70 f1* gene showed significant relative fold increases under elevated HP at 5 and 10°C. The *ldh* gene showed a significant relative fold decrease under elevated HP at 5°C. The *hsp70 f2* gene showed no significant RFC under elevated HP across the three temperatures (figure 1*a*). All six genes showed no significant change in expression between atmospheric and elevated HP at 27°C (figure 1*a*).

At atmospheric pressure (0.1 MPa), the *narg* gene showed no significant changes in expression across temperatures. The *cs* gene and the *hsp70 f1* gene showed significantly higher relative expression under atmospheric HP at 27°C in comparison to 5 and 10°C. In the *gapdh* gene, a significant fold increase was seen at 10 and 27°C in comparison to 5°C under atmospheric HP. Finally, significant relative fold decreases were observed in the *ldh* gene, and the *hsp70 f2* gene at 27°C in comparison to 5 and 10°C under atmospheric HP (figure 1*b*).

## 4. Discussion

For a strictly shallow-water eurytopic invertebrate, large variation in temperature or HP, in isolation, has effects on the transcriptional regulation of genes involved in distinct physiological mechanisms. Importantly, these effects are considerably more pronounced when temperature and HP act in combination [14]. Tested over a 168-h exposure, the current data show, for the first time, that the antagonistic effects of HP and temperature in response to acute experiments [14] are not merely artefacts of acute exposures. Therefore, these results have implications on our understanding of physiological limits to the depth distributions of marine organisms, and their ability to shift distribution ranges. The thermodynamics of volume change reactions indicate that decreasing temperature and increasing HP both favour reactions in which volumes decrease, and vice versa [13]. Volume change reactions are central to all biological processes, and the efficiency of such reactions has implications across an organism’s physiology [32]. If temperature and HP are considered a single entity (they covary throughout the marine biosphere) then all aquatic organisms exist within a specific thermodynamic envelope that is determined by their physiology. Outside this physiological scope, survival would either be time-limited or not possible [2]. Although ecological interactions are of clear importance in setting distribution limits, the physiological scope of an organism is likely to be of fundamental importance also [2,11]. When contemplating bathymetric migrations, the effects of the combination of temperature and HP must be considered. The complexity of understanding current range shifts and forecasting future shifts has been highlighted in several recent publications [10,33], and therefore an understanding of how combinations of stressors dictate physiological limits is important moving forwards.

The expression of the *cs* and *gapdh* genes increases with higher temperature at atmospheric pressure (figure 1*b*): such trends are expected and well understood, reflecting increasing metabolism with rising temperature [34]. However, under high HP, the trend is reversed with elevated *cs* and *gapdh* gene expression at lower temperature. The pattern of this gene expression may reflect changes in activity of their coding proteins [27]. Alternatively, changes in the expression of these markers may reflect an increase in ATP demand which cannot be met at high temperature when in combination with elevated HP. This may result in reduced scope for energetically costly transcriptional regulation. Although yet to be clearly resolved, a change in expression of these metabolic genes probably signifies some form of disturbance in key aerobic metabolism pathways. Increases in metabolism are well known as an important aspect of the CSR [18]. Under a combination of elevated HP and low temperature, increases in aerobic metabolism/metabolic demand, or diminished metabolic scope may therefore be a sign of increased stress. These inferences are corroborated by increased expression of the *hsp70 f1* gene under high HP and low temperature, another important aspect of the CSR indicating an increase in intracellular macromolecular damage [18]. The *ldh* gene, a marker of lactate accumulation and thus anaerobic metabolism [26], is downregulated where maximal expression of aerobic markers is observed. This may be a consequence of high cellular-level aerobic activity. The *narg* gene, a marker of pressure sensitivity associated with neurophysiological disturbances [14], shows the same trend under high HP conditions as the metabolic and CSR related genes, further inferring that the negative physiological effects of HP are greatest at low temperatures. The *narg* gene shows no change in regulation across temperatures at atmospheric HP, consistent with previous studies [14,21].

Overall, the regulation of transcriptional markers associated with aerobic metabolism, anaerobic metabolism, the CSR and pressure-specific intolerances indicate that the physiological effects of high HP or low temperature are exacerbated in combination with one-another, in line with thermodynamic theory. Equally, the effect of high HP is ameliorated at higher temperature. Our results corroborate previously published behavioural analysis over the same experimental exposures. Cottin *et al.* [15] demonstrated that at low temperature there was a reduction in locomotory activity under elevated HP in comparison to atmospheric HP. A reduction in locomotory activity coincided with an increase in the transcription of genes coding for HSP70 isoforms, suggesting that exposure to elevated HP and low temperature was stressful for the shrimp. Consequently, the observed reduction in locomotory activity may be a form of stress-induced energy conservation, a commonly observed response to stressful scenarios [35]. These data, considered alongside our observations of transcriptional regulation at 5°C, indicate that high HP at lower temperatures produces the greatest detrimental physiological effects for temperate marine ectotherms.

The transcriptional responses to HPs beyond current natural range limits observed in this study are similar to those recently documented in a mid-depth king crab species, *L. maja* [22]. Although further comparisons need to be made, this suggests that the response of *P. varians* to changing temperature and HP may be used to infer responses of other, more difficult to study, marine ectotherms. Although *P. varians* is an unlikely candidate for bathymetric migration under current conditions, it shares phylogenetic ancestry with deep-sea lineages [36] that may have undergone bathymetric range shifts from shallow-waters in the past [37]. As such we advocate *P. varians* as a useful experimental model in studies concerning HP and bathymetric range shifts where other marine ectotherms provide greater methodological and technological challenges in such laboratory-based physiological studies (such as the king crab, *L. maja* [22]).

Current data show that elevated HP induces the CSR, influences aerobic metabolic pathways, and induces pressure-specific physiological intolerances in a shallow-water ectotherm. These effects can be reduced by higher temperatures within the organisms’ thermal scope. Likewise, the effects of increasing HP are exacerbated by lower temperatures. It can be posited, from a purely physiological standpoint, that bathymetric migration down a warm isothermal water column is a physiologically viable alternative to latitudinal migration for marine ectotherms. By contrast, a cold isothermal water column may require inherent pressure tolerance or acclimatization/adaptation in order to overcome the effects of increasing HP. However, shallow-water cold-adapted ectotherms may have inherently higher HP tolerance due to low temperature adaptation [38]; this should be investigated further. Isothermal water columns currently exist at high latitudes and in some areas of enclosed seas, thus the potential for bathymetric migrations may be greatest there. Coincidentally, these are the same regions where latitudinal migrations are not possible. The more widespread stratified oceans, characterized by decreasing temperature with depth, may represent the greatest physiological challenge for shallow-water organisms attempting down-slope migration.

This study considers range limitation from a physiological standpoint, where in the natural environment ecological factors are of at least equal importance. However, an organism cannot survive where its physiology cannot sustain its life, and as such an understanding of physiological limits is an important precursor to combined eco-physiological studies. Future studies will benefit from an understanding of physiological limitations when combining ecological and physiological parameters, providing a more holistic understanding of species range dynamics. Our results demonstrate that temperature and HP are particularly significant environmental factors in combination and, as they covary throughout the ocean, it is important to consider them as acting concurrently rather than in isolation.

## Supplementary Material

Electronic supplementary material to; The potential for climate-driven bathymetric range shifts: sustained temperature and pressure exposures on a marine ectotherm, Palaemonetes varians The electronic supplementary material includes; qPCR normalisation strategies; evidence of qPCR assay optimisation for each gene studied; and the qPCR primer sequences and run conditions necessary to replicate the experiment

## Data accessibility

DNA sequences EMBL accession nos.: FR667656, LN713461, LN713462, GQ120565, FJ875280, FJ875279, FR667658, GQ120564. Primer sequences and qPCR assay optimization details are available in the electronic supplementary material.
