# Supplementary material for: The potential for climate-driven bathymetric range shifts: sustained temperature and pressure exposures on a marine ectotherm, Palaemonetes varians
Source: R Soc Open Sci. 2015 Nov 25;2(11):150472. doi: 10.1098/rsos.150472 (PMC4680618; doi:10.1098/rsos.150472)
Supplement: Electronic supplementary material to; The potential for climate-driven bathymetric range shifts: sustained temperature and pressure exposures on a marine ectotherm, Palaemonetes varians The electronic supplementary material includes; qPCR normalisation strategies; evidence of qPCR assay optimisation [file rsos150472supp1.docx]

**Electronic supplementary material to;**

**The potential for climate-driven bathymetric range shifts: sustained temperature and pressure exposures on a marine ectotherm, *Palaemonetes varians***

J. P. Morris^1, 2*^, S. Thatje^1^, D. Cottin^1^, A. Oliphant^1^, A. Brown^1^, B. Shillito^3^, J. Ravaux^3^, C. Hauton^1^

^1^Ocean and Earth Science, University of Southampton, European Way, Southampton SO14 3ZH, UK

^2^Current Address: Royal Belgian Institute of Natural Sciences, Rue Vautier 29, Brussels-1000, Belgium

^3^UPMC Université Paris 06, UMR-CNRS 7208, 7 Quai St-Bernard, F-75005 Paris, France

* jmorris@naturalsciences.be

**S1 - qPCR reference gene normalisation strategy**

In a pilot study, 4 candidate reference genes were tested by geNorm analysis using qBase+ software (Biogazelle, UK), and the geometric mean of the two commonly used reference genes (*rps26* and *rpl8*) (EMBL-EBI accession numbers; FR667658 and GQ120564) was determined as the best normalisation index against each gene of interest.

**S2 - qPCR assay optimisation**

**S2** Standard curves produced from qPCR cDNA dilution series for all genes studied. Graphs presented alongside: optimised primer concentrations (nM); dynamic linear range of the standard curve; linearity represented by an R^2^ value; and the amplification efficiency across the linear dynamic range.

**Supplementary table 1** qPCR primer sequences for each gene quantified

| **Gene** | **qPCR Primer** | **Sequence** | **Ref** |
| --- | --- | --- | --- |
| *narg* | FWD | AGA AAG TGC CAT GAA GTA GAT CGA (24) | [ESM1] |
|  | REV | AAC CTA AGC AGC TCA ACA TAA GAT CTT (27) |  |
| *rps26* | FWD | CGA CGT GAC AGG AAG ATT AGA ACT C (25) | [ESM1] |
|  | REV | ATG GGT TGC GTG GAC GTT (18) |  |
| *rpl8* | FWD | TCC CGG TCG TGG TGC ACC TAT T (22) | [ESM1] |
|  | REV | GAC GGC CTC GGT CAC CAG TCT TT (23) |  |
| *gapdh* | FWD | AAG GGC GCC GAG GTT GTT GCT GTA A (25) | [ESM1] |
|  | REV | GCA CCA GCC TTG CTC CAT GGA ATG T (25) |  |
| *hsp70 f1* | FWD | CCA GCC GTC ACC ATC CAG GTG T (22) | [ESM2] |
|  | REV | GCG GTC GAT GTC CTC CTT GCT G (22) |  |
| *hsp70 f2* | FWD | TTC CTG AGG AGG ATC GCA AA (20) | [ESM2] |
|  | REV | CAT TCC ACC AGG AGG AGC AC (20) |  |
| *cs* | FWD | CCT TGT CTG ATC CCT ACC TCT CA (23) | n/a |
|  | REV | CAT GCA ATG GTC CAG CAA GT (20) |  |
| *ldh* | FWD | TGG GAA TGA TGC CCT TGA A (19) | n/a |
|  | REV | GAA TCT CGC CTT TCC CTT GTC (21) |  |

**Table references**

ESM1. Morris JP, Thatje S, Ravaux J, Shillito B, Fernando D, Hauton C. 2015 Acute combined pressure and temperature exposures on a shallow-water crustacean: Novel insights into the stress response and high pressure neurological syndrome. *Comp Biochem Physiol A* **181**, 9-17.

ESM2. Cottin D, Shillito B, Chetemps T, Thatje S, Léger N, Ravaux J. 2010 Comparison of heat-shock responses between the hydorthermal vent shrimp *Rimicaris exoculata* and the coastal shrimp *Palaemonetes varians*. *J Exp Mar Biol Ecol* **393**, 9-16.
